# Internalization of the model minority myth and sociodemographic factors shaping Asians/Asian Americans' experiences of discrimination during COVID‐19

**DOI:** 10.1002/ajcp.12635

**Published:** 2022-11-28

**Authors:** Jacqueline Yi, Raymond La, B. Andi Lee, Anne Saw

**Affiliations:** ^1^ Department of Psychology University of Illinois at Urbana‐Champaign Champaign Illinois USA; ^2^ Department of Psychology DePaul University Chicago Illinois USA

**Keywords:** Asian Americans, COVID‐19, internalized oppression, model minority myth, vicarious discrimination

## Abstract

Despite appearing positive, the model minority myth (MMM), or the perception that Asian Americans are “problem‐free” minorities, maintains unfair racial hierarchies and discredits the pervasiveness of systemic racism faced by Asian Americans and other Black, Indigenous, and people of Color. This study investigated the role of internalized MMM in Asian/Asian Americans' (A/AA) experiences during the syndemic of COVID‐19 and our society's racial reckoning. Using a mixed methods approach, we analyzed A/AA college students' open‐ended responses to a query about their experiences as A/AA during COVID‐19, which resulted in qualitative themes of Personal and Vicarious Discrimination, Vigilance, Safety due to Ethnicity, Safety due to Environment, and No Difference during COVID‐19. We then conducted a series of logistic and linear regression models to examine how internalized MMM and sociodemographic factors (i.e., ethnic group, gender, and generational status) were associated with qualitative themes and quantitative measures of COVID‐related discrimination. Overall, findings demonstrated that greater internalized MMM, as well as identifying as South Asian, male, and an international/first‐generation immigrant student, were linked to fewer qualitative and quantitative reports of vicarious discrimination. We conclude with implications for research and practice in community psychology that further examine the racialized experiences among A/AA college students and ultimately seek to challenge the MMM and racial hierarchies perpetuating systems of oppression.

## Highlights

- This mixed‐methods study examines Asians/Asian Americans' experiences of discrimination during COVID‐19.
- We hypothesized that internalization of the model minority myth (MMM) is a form of internalized oppression that obscures COVID‐related, anti‐Asian racism.
- Greater internalized MMM was linked to less reports of vicarious discrimination during the pandemic.
- Reports of discrimination and vigilance also differed by Asian ethnic group, gender, and generational status.

## INTRODUCTION

Since the COVID‐19 pandemic began, Asians/Asian Americans (A/AA) have reported significantly greater racial discrimination, with incidents occurring with even higher frequency one year later (Chen et al., 2020; Jeung et al., 2021; Misra et al., 2020). The distinct experiences of A/AA during COVID‐19 reveal a syndemic of interrelated, synergistic threats—including COVID‐19 illness and death, anti‐Asian racism, social and economic stressors, and health and mental health challenges (Grills et al., 2022; Saw et al., 2022). A/AA college students are at a particularly challenging intersection of the syndemic, as they experienced various disruptions in their schooling, work, and housing, as well as faced racial tensions on campus and a lack of support from institutional leaders (Molock & Parchem, 2021; Tausen et al., 2020; Wong‐Pandoongpatt et al., 2022). Extant research has documented the negative psychological consequences of anti‐Asian discrimination, including increased anxiety, depression, and posttraumatic stress disorder symptoms among A/AA (Hahm, Ha, et al., 2021; Lee & Waters, 2021). However, a deeper examination of A/AA's experiences of personal and vicarious discrimination (i.e., indirect race‐related stress when witnessing or learning about discrimination experienced by someone else in one's racial group; Harrell, 2000) during COVID‐19 is needed.

Studying Black, Indigenous, and people of Color (BIPOC) experiences of discrimination is an integral part of community psychology and critical in promoting the field's values of antiracism and social justice (Bernal et al., 2020). Yet, compared with other racial minorities, the racialized experiences of A/AA are often overlooked in community psychology. Thus, the current study addresses this gap by highlighting ways to better understand A/AA college students’ experiences amidst the syndemic of COVID‐19 and racism. In this study, we also aim to contribute to the literature on how A/AA's racial attitudes, specifically their internalization of the model minority myth (MMM), may shape their experiences during COVID‐19.

### Asian American stereotypes and discrimination

To fully understand anti‐Asian discrimination during COVID‐19, we must examine racial stereotypes within the context of the United States. These stereotypes have a long history of reinforcing anti‐Asian racism, operate at structural, cultural, and individual levels (Tseng & Lee, 2021; Williams et al., 2019), and are often internalized by A/AA themselves (Yoo et al., 2010).

The MMM portrays A/AA as a minority group that achieved social mobility because of their educational prowess and work ethic (Chou & Feagin, 2015). Although the MMM is seemingly positive, scholars assert that the MMM is dialectical to the “yellow peril” stereotype, which portrays A/AA as perpetual foreigners invading the United States and threatening White American society (Okihiro, 2014). The stereotype characterizes A/AA as uncivilized and unhygienic, and consequently has been used to scapegoat A/AA for disease outbreaks such as the bubonic plague (Chen et al., 2020) and justify laws that systematically discriminated against A/AA (Nagata et al., 2019). Even though the “yellow peril” stereotype contradicts the MMM, some suggest that the MMM repackages anti‐Asian xenophobia endorsed by the “yellow peril” stereotype in a socially acceptable manner (Li & Nicholson, 2021).

The MMM and “yellow peril” stereotypes place A/AA in a unique position in America's racial hierarchy, commonly explained through racial triangulation theory (C. J. Kim, 1999). Racial triangulation posits that A/AA is valorized as a relatively successful minority group and socially ranked above Black Americans as “honorary Whites.” Yet, the “yellow peril” stereotype prohibits A/AA from being socially accepted as “American” compared with Black and White counterparts. This relative positioning has several consequences. First, it falsely implies that A/AA do not experience health disparities despite empirical research suggesting otherwise (Ibaraki et al., 20142014). Furthermore, it obscures the structural inequalities impacting A/AA (Xu & Lee, 2013), resulting in decreased funding, research, and policies targeting A/AA health disparities (J. H. J. Kim et al., 2021), and further reinforcing racialized stereotypes and structural inequalities (Saw et al., 2022). Finally, scholars suggest that the MMM was developed to criticize other racial minorities and justify racial inequality by highlighting a fabricated exception (Sakamoto et al., 2012). In sum, the MMM not only promotes a racial divide but also allows plausible deniability of inequalities experienced by both A/AA and other BIPOC communities.

### Internalized MMM and discrimination

Although the MMM negatively impacts A/AA and other BIPOC, some A/AA internalize the MMM as part of their self‐identity because of its seemingly positive connotations (Yoo et al., 2010). Internalizing the MMM requires one to believe that they can achieve social mobility through hard work because they are unaffected by racism (Yoo et al., 2010), and may result in adopting attitudes associated with the myth of white supremacy, such as the denial of racial barriers and inequalities (Yi & Todd, 2021). Previous studies have also demonstrated links between internalized MMM and negative psychological outcomes, such as greater psychological distress and decreased mental health help‐seeking behaviors (Atkin et al., 2018; P. Y. Kim & Lee, 2014). However, no study to our knowledge has examined how internalized MMM may impact A/AA's experiences of racial discrimination, which is especially relevant during the syndemic when reported exposures to anti‐Asian racial discrimination conflict with MMM beliefs. Therefore, additional research is needed to understand how they experience and respond to racial discrimination, especially during a racial reckoning that may challenge their internalized beliefs.

### Racial discrimination during COVID‐19

#### Personal and vicarious discrimination

One factor that may be relevant in understanding how A/AA experience COVID‐related racism is whether they personally or vicariously (i.e., indirectly) experienced discriminatory incidents. Although studies typically document the negative effects of personal discrimination, vicarious discrimination may also be harmful to BIPOC because it transmits racial trauma and pervasive stress to a broader community (Heard‐Garris et al., 2018). Therefore, the examination of personal discrimination does not fully capture A/AA experiences of racial discrimination. Indeed, multiple studies found that A/AA more commonly experienced vicarious discrimination compared to personal discrimination during the COVID‐19 pandemic, indicating a need to further investigate the potential impact of vicarious discrimination (Chae et al., 2021; Hahm,  Xavier Hall, et al., 2021). Lee and Waters (2021) also found that A/AA adults reported social media and reading news stories as common sources of experiencing vicarious discrimination. Vicarious discrimination and its accompanying race‐related stress negatively impact A/AA well‐being. Across both A/AA adults and Chinese American adolescents, vicarious racism during COVID‐19 was related to greater endorsement of depression and anxiety symptoms (Chae et al., 2021; Zong et al., 2021). In sum, extant literature illustrates the significance of vicarious discrimination in understanding A/AA's experiences during the syndemic.

However, it is uncertain how A/AA experience vicarious discrimination and the potential implications. On the one hand, both personal and vicarious racial discrimination may produce cognitive dissonance among those who internalize the MMM, because they must confront the reality that their racial group is not exempt from racial discrimination (Chou & Feagin, 2015). On the other hand, vicariously learning how others experience racial discrimination allows plausible deniability of this reality. Therefore, additional research is needed to understand A/AA experiences of racial discrimination among those who internalized the MMM. Specifically, both personal and vicarious discrimination need to be examined to fully understand how the MMM shapes responses to racial discrimination.

#### Sociodemographic factors of discrimination

Sociodemographic factors, such as ethnic group membership, may also affect how A/AA experience discrimination during the syndemic. As previously noted, A/AA of various ethnic backgrounds reported increased discrimination during COVID‐19 (Chen et al., 2020; Misra et al., 2020), although Chinese individuals comprise a larger portion of reports sent to Stop AAPI Hate (Jeung et al., 2021). However, Woo and Jun (2021) reported that Chinese Americans and other Asian Americans do not statistically differ in how racial discrimination may affect their depressive symptoms. Although this finding suggests that the syndemic is relevant to A/AA beyond those with a Chinese background, it also raises questions about whether these findings reflect the targeting of non‐Chinese A/AA due to phenotypic similarity or the racialized attribution of the COVID‐19 pandemic to A/AA. Relatedly, no study to our knowledge examined Asian ethnic group differences in COVID‐related personal and vicarious discrimination. This literature gap is notable because opportunities to experience personal and vicarious discrimination may be dependent on factors tied to ethnic groups. Therefore, additional research is needed to determine whether ethnic group differences exist in personal and vicarious experiences of discrimination and the potential factors driving these differences.

Gender may also be a factor affecting COVID‐19‐related discrimination. According to Stop AAPI Hate, women made up 64.8% of all their reports, and gender was noted as a common motivating factor for discrimination (Jeung et al., 2021). Although most studies included gender as a covariate and did not interpret related findings, theoretical frameworks in feminist psychology (Lewis, 2018) and Asian American studies (Tajima, 1989) provide insight into why A/AA women and gender minorities experience more racial discrimination. For example, Lewis’ (2018) psychosocial model of gendered racism highlights how women of Color such as A/AA women experience covert and overt sexism and racism in their everyday lives. This model aligns with Tajima's (1989) assertion that Asian American women are portrayed as hypersexualized and objectified by the (White) male gaze (Kim & Chung, 2005). Additionally, Endo's (2021) autoethnography on processing the 2021 Atlanta Massacre illustrates how A/AA women may experience heightened vigilance and trauma from vicarious discrimination due to gendered Orientalism associated with attacks such as the Atlanta Massacre.

Finally, generational status may also impact discriminatory experiences during the syndemic. Previous research indicates that racial discrimination's frequency and impact on health increase with time in the United States for A/AA immigrants (Yoo et al., 2010). Yet research on how generational status impacts racial discrimination experiences is mixed. For example, Alamilla et al. (2017) found a positive relationship between generational status and discrimination in a multiethnic A/AA sample, while Juang and Cookston (2009) found that among Chinese Americans, immigrants reported more discrimination than their US‐born counterparts. Liu et al. (2020) also found that second and third‐generation immigrants experienced significantly more COVID‐19‐related racial discrimination compared with first‐generation immigrants in a national sample of BIPOC. No studies to date have examined generational status specifically among A/AA's discriminatory experiences during COVID‐19.

### Current study

In the current study, we utilized a mixed methods approach to examine how internalized MMM and sociodemographic factors shape A/AA's experiences of discrimination during COVID‐19. We engaged in qualitative analysis and developed themes around A/AA college students' open‐ended reflections on their experiences during COVID‐19. We then tested whether internalized MMM, ethnic group, gender, and generational status were linked to the endorsement of qualitative themes, along with quantitative variables on personal and vicarious discrimination. We hypothesized that greater internalized MMM will be associated with less qualitative and quantitative reports of discrimination faced by the Asian American community amidst the pandemic. Given that A/AA ar not a monolith and diverse discriminatory experiences exist across Asian subgroups (Chou & Feagin, 2015), we also hypothesized that there would be differences by ethnic group, gender, and generational status. Taken together, the current study aimed to address gaps in the literature on personal and vicarious experiences of discrimination among A/AA during COVID‐19 and their associations with sociodemographic factors and internalized MMM.

## METHODS

### Participants and procedures

From September 2020 to November 2021, we collected data from a large Midwestern university's Psychology departmental subject pool, which consisted of undergraduate students enrolled in introductory psychology courses. The mean age of the sample was 18.9 (*SD* = 1.3). Students from various majors (e.g., psychology, biology, computer science, undecided) who identified as “Asian” in a prescreening survey responded to an online battery of measures as part of a larger university Institutional Review Board‐approved study on sociopolitical attitudes. The survey took approximately 50 minutes to complete, and students received course credit for completion. Our final sample for the current study consisted of 314 A/AA respondents. We checked for missing data on all study variables and found that no respondent missed more than one item on each scale. Using available item analysis (Parent, 2012), we did not drop any respondents, as only missing one item on the scales resulted in at least 80% or more of the items completed.

Students identified their ethnicity as Chinese (39.8%), Indian (23.3%), Korean (15.3%), Filipino/a/x (4.5%), Pakistani (4.1%), Vietnamese (2.9%), Japanese (1.6%), Sri Lankan (0.6%), Bangladeshi (0.6%), Thai (0.3%), Laotian (0.3%), Burmese (0.3%), and Malaysian (0.3%). There were also students who identified with multiple Asian ethnicities (6.9%; e.g., Chinese and Vietnamese). We further categorized ethnicity into the following regional ethnic groups: East Asian (58.3%; e.g., Chinese, Korean, multiethnic Chinese and Korean), South Asian (29.6%; e.g., Indian, Sri Lankan, multiethnic Indian and Pakistani), Southeast Asian (8.9%; e.g., Filipinx, Cambodian, multiethnic Filipinx, and Vietnamese), and multiethnic across regional groups (3.2%; e.g., Chinese and Filipinx). Respondents identified as women (58.9%), men (39.5%), nonbinary (0.6%), trans women (0.3%), trans men (0.3%), and genderqueer/gender nonconforming (0.3%). We categorized gender to examine differences among women, men, and gender minorities, such that nonbinary, trans, and genderqueer/gender nonconforming students into one category. For generational status, 44% identified as international students and/or first‐generation immigrants (i.e., not born in the United States), 52.5% identified as second‐generation (i.e., born in the United States, parents not born in the United States), 3.2% identified as third generation (i.e., born in the United States, parents born in the United States, grandparents not born in the United States), no respondents identified as the fourth generation, and 0.3% identified as fifth generation or higher. In our analyses, we categorized generational status into international/first‐generation immigrant students, second‐generation students, and third‐ or higher generation students. Of note, a majority of respondents in the international/first‐generation category reported that they were international students (74.2%). Among international students, 89.5% identified as East Asian, 9.3% South Asian, and 1.2% Southeast Asian, with no international students identifying as multiethnic across regional groups. a total of 54% of international students identified as women, 44.8% as men, and 1.2% as gender minorities.

### Measures

#### Internalized MMM: Unrestricted Mobility

We used the Internalized MMM Measure's Unrestricted Mobility subscale (Yoo et al., 2010) to assess A/AA's attitudes toward their own racial group as less likely to experience discriminatory barriers, compared with other racial minorities. This subscale consists of five items on a Likert‐type scale ranging from 1 (*strongly disagree*) to 7 (*strongly agree*) that measure attitudes toward Asian Americans as overcoming barriers of discrimination (e.g., “Asian Americans are less likely to encounter racial prejudice and discrimination”; “Asian Americans are more likely to be treated as equals to European Americans”). Previous studies demonstrated good internal consistency (P. Y. Kim & Lee, 2014; Parks & Yoo, 2016), and greater scores on the unrestricted mobility subscale are associated with lower psychological well‐being (Atkin et al., 2018) and greater colorblindness (Yi & Todd, 2021). In the current study, *α* = .82.

#### Experiences of discrimination during COVID‐19

Adapted from the Schedule of Racist Events (Landrine & Klonoff, 1996), we developed eight items assessing A/AA's personal experiences of discrimination during COVID‐19 (e.g., subjected to offensive comments, being physically hurt, others avoided contact with you, threatened or harassed because of your race/ethnicity). We also developed one item to assess the frequency of vicarious discrimination: “How many times have you witnessed racial/ethnic discrimination directed toward A/AA people (e.g., in‐person, on social media, and in the news)?” Students rated the frequency of these experiences on a scale from 1 (*never happened*) to 6 (*happened almost all the time*). For personal experiences of discrimination, we created a mean of scale items, and internal consistency was high (*α* = .88). Finally, we asked the open‐ended question, “What has been your experience as an Asian/Asian American person during COVID‐19?” and responses were coded for themes.

### Mixed methods approach

Using a mixed methods approach, themes identified from our qualitative data were examined as frequency counts and analyzed with quantitative measures of internalized MMM, personal and vicarious discrimination during COVID‐19, and other constructs such as Asian regional ethnic group, gender, and generational status. Assigning numerical values to nonnumerical data, or “quantizing,” is a form of conversion mixed data analysis and allows for rich conversation between qualitative and quantitative data (Sandelowski et al., 2009; Teddlie & Tashakkori, 2006). Our mixed methods purposes were (1) *expansion*, where we sought to extend the scope and breadth of inquiry by using qualitative and quantitative methods to assess different phenomena (i.e., A/AA college students’ experiences during COVID‐19 and internalized MMM, respectively); and (2) *triangulation*, where we sought convergence in our findings on qualitative themes and quantitative variables (Greene, 2007). Specifically, we tested whether internalized MMM was associated with qualitative themes of personal and vicarious discrimination and quantitative measures of personal and vicarious discrimination in similar or different ways.

The present study was informed by the research paradigms of constructivism‐interpretivism and critical‐ideological theories (Kincheloe & McLaren, 2000; Schwandt, 1994). Both paradigms emphasize understanding people's lived experiences and consider how there are multiple realities shaped by people's unique social contexts, but critical‐ideological theory further articulates the goal of attending to power, privilege, and oppression and challenging systemic inequality (Creswell, 1998). These paradigms align with the values of community psychology (Banyard & Miller, 1998) and fit with the current study's examination of anti‐Asian discrimination and the role of the MMM in the lives of A/AA college students. We also reflected on and discussed our biases, values, and sociopolitical commitments throughout the research process (Greene, 2007). For example, we considered how our social locations as East/Southeast Asian community psychologists affected our expectations and interpretations of the data, such that we anticipated differences in experiences with anti‐Asian discrimination related to COVID‐19 between East and South Asian students. We also expected some students to deny or minimize the existence of anti‐Asian discrimination and uphold the MMM. From our critical‐ideological stance, we were ultimately driven by our desire to challenge systems of oppression that perpetuate anti‐Asian racism and xenophobia with this research.

Our analysis began with deriving themes from open‐ended responses using a modified consensual qualitative research approach (CQR‐M; Spangler et al., 2012). CQR‐M utilizes large samples and brief qualitative data and provides frequency labels based on theme endorsement (Ladany et al., 2012). We developed thematic categories, independently coded the data for the presence or absence of themes, and met to discuss coding until reaching a consensus. We then examined associations among endorsement of qualitative themes and quantitative variables. As part of our preliminary analyses, we also examined differences in internalized MMM by ethnic group, gender, and generational status using analysis of variances (ANOVAs). Next, we conducted a series of logistic regression models to investigate how sociodemographic factors and internalized MMM are linked to the presence or absence of qualitative themes. We first examined a model to test associations between endorsement of themes and ethnic group, gender, and generational status (Model 1). We then examined a model that only tested associations between themes and internalized MMM (Model 2). We then examined a model that included all variables (Model 3). Finally, we conducted a series of linear regression models to test how these variables might be associated with quantitative measures of COVID‐related discrimination, using the same structure as Models 1–3 in our logistic regression analyses.

## RESULTS

### Qualitative themes

Various themes were identified from A/AA college students' responses to the open‐ended question about their experiences during COVID‐19 (see Table 1). The theme No Difference, endorsed by 26.3% of the sample, demonstrated how many students perceived no change or impact of COVID‐19 as A/AA people. For example, several students briefly responded “Nothing” or “Normal,” and some students explicitly stated, “Equal to all races” and “I have not felt discrimination against my race.” One student wrote: “I have rarely experienced racism as I grew up and it has not changed since COVID‐19.” Responses coded with this theme also included students stating that their experiences were “Fine,” “Good,” or “Okay.”

**Table 1:** Themes from Asian/Asian American students’ open‐ended responses on experiences during COVID‐19

Theme	Definition	Example Quotes	*f*	%
1. No Difference	Stated that nothing has changed or there was no impact of COVID‐19 as an Asian/Asian American person	“Nothing. Same as usual”; “Fine”; “No different from anyone else. Nobody has thrown racial insults my way or towards my family.”	83	26.3
2. Safety due to Environment	Felt safe because of the environment they are living in, such as an Asian country or a neighborhood open to diversity	“I'm in China, so there's no discrimination here.”; “My neighborhood is also pretty accepting and open‐minded, so I experienced very little discrimination or malicious attitudes.”	33	10.4
3. Safety due to Ethnicity	Cited their ethnicity, mainly identifying as South Asian, as the reason for feeling safe during COVID‐19	“I am South Asian, so the prejudice against Asian Americans didn't apply to me”; “As someone who is of Indian ethnic background, I was not affected as negatively as I know my other East Asian friends were. It has generally been the same for me.”	31	9.8
4. Personal Discrimination	Directly experienced anti‐Asian discrimination during COVID‐19 (e.g., being assumed to have COVID‐19, stared at, avoided in public)	“Receiving looks from people who thought I had COVID simply because of my race”; “Some people put their masks on or walk away faster from me and my group of Asian friends and I experienced it on campus.”	43	13.6
5. Vicarious Discrimination	Indirectly experienced anti‐Asian discrimination during COVID‐19 (e.g., witnessing racism directed towards other Asian/Asian Americans)	“Although I did not experience anything firsthand, my mom has told me of a time where someone changed their course of direction to avoid her. I have also heard many stories on social media as well as from friends/acquaintances”; “I've seen a lot of news about Asians being attacked and posts on social media bashing Asians and it's made me very sad and angry.”	96	30.4
6. Vigilance	Felt tense or worried about being targeted as an Asian/Asian American person during COVID‐19	“Terrifying. I was scared to leave the house”; “I sometimes find myself in public worrying that people may treat me with disdain because they imagine I have COVID due to myself being an Asian.”	40	12.7

*Note*: *f* is the frequency of theme endorsement; % is the percent of endorsement out of 314 respondents.

The theme Safety due to Environment, endorsed by 10.4% of the sample, reflected how some students reported feeling safe as an A/AA person during COVID‐19 specifically because of their context, such as residing in an Asian country, a community with many A/AA people, or a neighborhood with an openness to diversity. For example, one student wrote: “I am fortunate to live in a diverse community. Therefore, I had very limited racial aggression toward me compared with my other Asian friends in other communities. These friends were told to go back to where they belong and that it was their fault for COVID‐19.” The theme Safety due to Ethnicity, endorsed by 9.8% of the sample, demonstrated how some students cited that their ethnicity, mostly those identifying as South Asian, was the reason for not being targeted during the pandemic. Several students endorsing this theme elaborated on how A/AA of East Asian descent were primarily targeted. One student wrote: “As an Indian American person, I did not experience much of a difference as far as how I am treated in society, as the East Asians and those who are visually similar to these groups experienced a majority of the hate and discrimination.”

In contrast, the theme of Personal Discrimination, endorsed by 13.6% of the sample, revealed accounts of students’ firsthand experiences of anti‐Asian discrimination during COVID‐19. Students reported that they were subjected to offensive comments and assumed to have COVID‐19 because they were A/AA (e.g., “Someone cursed me because I'm Chinese”; “During work, I was asked by a customer if I had the virus solely based off the fact that I was Asian”; “Lots of people in my hometown asking me if I brought COVID to the United States. after I visited Korea over the summer”). Students also reflected on their experiences of being stared at or avoided contact with in public because they were A/AA (e.g., “It has spiked significantly where people make obviously disgusted faces when I am at the store”; “People tend to look at me weirdly and move away when I sneeze in public, despite wearing a facemask like them”; “People avoided my family in public because of our race”).

The theme Vicarious Discrimination, endorsed by 30.4% of the sample, demonstrated how many students witnessed anti‐Asian discrimination directed toward other members of their racial group, such as hearing about the experiences of A/AA friends or family members. One student wrote: “My family talks about some people calling them out for being Asian and having COVID or being slightly dangerous because of the virus. There was a time when my dad was told to leave a grocery store.” Another student wrote: “An Asian American friend of mine had an egg thrown in his direction and it was a wake‐up call that this could happen to any of us Asian Americans at any time.” Many students also expressed feeling fearful, sad, angry, stressed, and disheartened as they witnessed anti‐Asian discrimination through social media and online platforms. One student wrote: “I have been home all the time so most of my interaction with the world is through social media. I have seen Chinese and other Asian Americans being harassed, threatened, and even assaulted because of their race which greatly stresses me.” Another student wrote: “It has definitely been difficult to see all the hate Asians have been receiving on and off social media in relation to the pandemic. It's very hard to see people of your kind being discriminated against.” Responses coded with this theme also included students hearing racist rhetoric directed toward A/AA as an entire racial group. One student wrote: “Seeing Trump and his supporters call COVID the ‘China Virus’ is extremely offensive, it made me sad to see Asians receiving racism (like being harassed, Chinese restaurants went out of business because people were afraid of getting COVID).”

The theme Vigilance, endorsed by 12.7% of the sample, reflected A/AA students' fear and anticipation of personal and vicarious discrimination. Students reported feeling worried and scared about personal victimization and the safety of their friends and family. One student wrote: “I personally didn't experience any discrimination, but I became hyper‐aware of my surroundings because I was afraid that I might.” Another student wrote: “For an Asian American living in the suburbs, I was mostly scared for my parents who have to go to work in the city all day. In the news, you heard of people bashing Asians because of COVID‐19, and my parents who have to walk out in the streets vulnerable, it was kind of stressful.” Students also reported persistent self‐monitoring in their environment or avoidance of places where racial discrimination could occur. One student wrote: “My hometown is predominantly Caucasian, so I felt that all eyes were on me when I did menial tasks like going to the grocery store. If I needed to sneeze, I forced it away because I knew what others would immediately assume.” Another student wrote: “It's been really bad… I have reconsidered going to college in the States. The discrimination here is very serious.”

### Associations among qualitative themes and quantitative variables

To examine associations among qualitative themes, we calculated *φ* coefficients, where positive coefficients indicate that the presence of one theme increased the likelihood of another theme (see Table 2). Out of 15 possible pairs of themes, we found significant associations among six pairs. Associations were in the expected direction; for example, the themes No Difference and Personal Discrimination were negatively associated. We also examined point‐biserial correlations between qualitative themes and quantitative variables. Higher scores on the personal discrimination scale were significantly associated with a greater likelihood of endorsing the theme Personal Discrimination and a lower likelihood of endorsing the themes No Difference and Safety due to Environment. Higher scores on the vicarious discrimination item were significantly associated with a greater likelihood of endorsing the themes Personal Discrimination, Vicarious Discrimination, and Vigilance and a lower likelihood of endorsing the theme No Difference.

**Table 2:** Dependence of qualitative themes and quantitative variables

Theme/variable	1	2	3	4	5	6	7	8	9
1. No Difference	_	−0.11	−.17*	−.24*	−.39*	−.23*	−.20*	−.28*	0.04
2. Safety due to Environment		_	−0.04	−0.12	0.02	−0.07	−.16*	−0.06	−0.04
3. Safety due to Ethnicity			–	−0.10	−0.03	−.13*	0.04	0.03	0.09
4. Personal Discrimination				–	−0.04	0.07	.17*	.1*	0.03
5. Vicarious Discrimination					_	.31*	0.07	.24*	−.13*
6. Vigilance						_	0.03	.13*	0.03
7. *Personal discrimination scale*							_	.47*	−0.06
8. *Vicarious discrimination item*								_	−.20*
9. *Internalized MMM*									

*Note*: *φ* coefficients are used to show the strength of dependence among qualitative themes. A positive sign shows that the presence of one theme increases the likelihood of the presence of the second theme, whereas a negative sign shows that the presence of one theme decreases the likelihood of the presence of the second theme. Quantitative variables are italicized. Point‐biserial correlation coefficients are used to show the strength of dependence between quantitative variables and qualitative themes.

* *p* < .05.

### Preliminary analyses

We also tested for differences in internalized MMM by ethnic group, gender, and generational status using ANOVAs (see Table 3). There were no significant differences by ethnic group and generational status. There were significant gender differences, such that women had significantly lower internalized MMM unrestricted mobility beliefs compared with men.

**Table 3:** ANOVA results by ethnic group, gender, and generational status for internalized MMM

	*M (SD)*	*M (SD)*	*M (SD)*	*M (SD)*	*F*	Tukey post hoc tests
**Ethnic Group**	**East Asian**	**South Asian**	**Southeast Asian**	**Multi‐Ethnic**		
Internalized MMM	3.31 (1.04)	3.18 (1.20)	3.11 (1.22)	2.84 (1.30)	0.91	–
**Gender**	**Men (M)**	**Women (W)**	**Gender Minorities**			
Internalized MMM	3.49 (1.10)	3.06 (1.08)	3.72 (1.57)		6.06*	W < M
**Generational Status**	**International/First Gen**	**Second Gen**	**Third+ Gen**			
Internalized MMM	3.23 (1.00)	3.24 (1.19)	3.45 (1.29)		0.21	–

*Note*: Tukey post hoc tests control type I error at *α* = .05 for the pairwise tests for each significant ANOVA.

Abbreviations: ANOVA, analysis of variance; Gen, generation; MMM, model minority myth.

* *p* < .05.

### Links between qualitative themes, sociodemographic factors, and internalized MMM

We then conducted a series of logistic regression models to examine how internalized MMM, as well as ethnic group, gender, and generational status, were linked to whether A/AA endorsed qualitative themes (see Table 4). We did not test the themes Safety due to Environment and Safety due to Ethnicity in the logistic regression models due to low endorsement (i.e., less than 40 respondents endorsed themes). In Model 1, we tested if there were demographic differences in the endorsement of themes. Results demonstrated that South Asian students, compared with East Asians, were significantly more likely to endorse the theme No Difference and significantly less likely to endorse the themes Personal Discrimination, Vicarious Discrimination, and Vigilance. Compared with men, women and gender minorities were significantly more likely to endorse Vicarious Discrimination, and women were significantly more likely to endorse Vigilance. There were also significant differences by generational status. Compared with international/first‐generation students, second‐generation students were significantly more likely to endorse the themes Personal Discrimination, Vicarious Discrimination, and Vigilance. In Model 2, we tested the main effect of internalized MMM on the endorsement of qualitative themes. We found that greater internalized MMM significantly was associated with lower endorsement of Vicarious Discrimination. Internalized MMM was not significantly associated with the endorsement of any other qualitative themes. In Model 3, we tested if internalized MMM was linked to qualitative themes over‐and‐above ethnic group, gender, and generational status. We did not find that internalized MMM was significantly related to any themes over‐and‐above demographic variables.

**Table 4:** Associations between qualitative themes and quantitative variables and ethnic group, gender, generational status, and internalized MMM

	**No difference** *B* (*SE*) *OR* [95% CI]	**Personal discrimination** *B* (*SE*) *OR* [95% CI]	**Vicarious discrimination** *B* (*SE*) *OR* [95% CI]	**Vigilance** *B* (*SE*) *OR* [95% CI]	**Personal discrimination** Scale *B* (*SE*)	**Vicarious discrimination** Item *B* (*SE*)
**Model 1**						
South Asian	1.03* (0.31)	−1.70* (0.52)	−1.89* (0.34)	−1.86* (0.53)	0.14 (0.11)	−0.19 (0.18)
	2.81 [1.53, 5.14]	0.18 [0.07, 0.51]	0.32 [0.16, 0.62]	0.16 [0.06, 0.44]		
Southeast Asian	0.14 (0.51)	−0.53 (0.60)	−0.31 (0.46)	−1.08 (0.67)	0.01 (0.17)	0.08 (0.27)
	1.15 [0.42, 3.12]	0.59 [0.18, 1.90]	0.73 [0.30, 1.82]	0.34 [0.09, 1.27]		
Multiethnic Asian	−0.53 (1.09)	−1.18 (1.10)	1.50 (0.80)	−0.97 (1.11)	0.12 (0.27)	0.19 (0.44)
	0.59 [0.07, 4.97]	0.31 [0.04, 2.67]	4.48 [0.94, 21.29]	0.38 [0.04, 3.33]		
Women	−0.49 (0.27)	0.47 (0.36)	0.99* (0.29)	0.83* (0.40)	−0.00 (0.09)	0.40* (0.15)
	0.62 [0.36, 1.05]	1.61 [0.79, 3.26]	2.70 [1.52, 4.79]	2.28 [1.05, 4.95]		
Gender minorities	−0.74 (1.15)	0.91 (1.20)	2.06* (0.97)	1.15 (1.22)	−0.24 (0.37)	0.66 (0.60)
	0.48 [0.05, 4.54]	2.49 [0.24, 25.93]	7.86 [1.18, 52.35]	3.16 [0.29, 34.63]		
Second generation	−0.50 (0.29)	0.79* (0.37)	1.22* (0.30)	1.27* (0.40)	−0.03 (0.10)	0.19 (0.16)
	0.61 [0.35, 1.08]	2.20 [1.06, 4.58]	3.37 [1.86, 6.10]	3.57 [1.64, 7.77]		
Third+ generation	−1.52 (1.09)	1.36 (0.78)	−0.46 (0.90)	_	−0.29 (0.26)	−0.35 (0.42)
	0.22 [0.03, 1.86]	4.30 [0.93, 19.90]	0.63 [0.11, 3.66]			
**Model 2**						
Internalized MMM	0.09 (0.11)	0.07 (0.15)	−0.25* (0.11)	0.09 (0.15)	−0.04 (0.04)	−0.24* (0.07)
	1.09 [0.87, 1.37]	1.07 [0.80, 1.42]	0.78 [0.62, 0.97]	1.09 [0.81, 1.47]		
**Model 3**						
South Asian	1.04* (0.31)	−1.69* (0.52)	−1.19* (0.34)	−1.85* (0.53)	0.13 (0.11)	−0.22 (0.18)
	2.83 [1.54, 5.19]	0.18 [0.07, 0.51]	0.30 [0.16, 0.60]	0.16 [0.06, 0.45]		
Southeast Asian	0.14 (0.51)	−0.51 (0.60)	−0.37 (0.47)	−1.04 (0.67)	−0.00 (0.17)	0.04 (0.27)
	1.16 [0.42, 3.15]	0.60 [0.19, 1.94]	0.69 [0.27, 1.74]	0.35 [0.10, 1.32]		
Multiethnic Asian	−0.49 (1.09)	−1.15 (1.10)	1.38 (0.80)	−0.91 (1.12)	0.10 (0.27)	0.07 (0.43)
	0.61 [0.07, 5.21]	0.32 [0.04, 2.76]	3.99 [0.82, 19.27]	0.40 [0.05, 3.56]		
Women	−0.45 (0.28)	0.50 (0.37)	0.92* (0.30)	0.86* (0.40)	−0.02 (0.10)	0.31* (0.15)
	0.64 [0.37, 1.09]	1.64 [0.80, 3.36]	2.50 [1.40, 4.47]	2.36 [1.08, 5.15]		
Gender minorities	−0.77 (1.16)	0.92 (1.19)	2.15* (0.99)	1.15 (1.22)	−0.23 (0.37)	0.70 (0.59)
	0.46 [0.05, 4.46]	2.50 [0.24, 25.84]	8.58 [1.24, 59.39]	3.15 [0.29, 34.06]		
Second generation	−0.50 (0.29)	0.78* (0.37)	1.24* (0.30)	1.26* (0.40)	−0.02 (0.10)	0.21 (0.16)
	0.61 [0.34, 1.07]	2.19 [1.05, 4.56]	3.46 [1.90, 6.29]	3.54 [1.62, 7.71]		
Third+ generation	−1.55 (1.09)	1.45 (0.78)	−0.39 (0.90)	_	−0.28 (0.26)	−0.27 (0.42)
	0.21 [0.03, 1.82]	4.24 [0.92, 19.65]	0.68 [0.12, 3.99]			
Internalized MMM	0.07 (0.12)	0.06 (0.16)	−0.22 (0.12)	0.10 (0.16)	−0.04 (0.04)	−0.22* (0.07)
	1.07 [0.85, 1.36]	1.06 [0.78, 1.44]	0.80 [0.63, 1.02]	1.11 [0.80, 1.53]		

*Note*: No third+  generation students endorsed the theme of Vigilance.

Abbreviations: CI, confidence interval; MMM, model minority myth; OR, odds ratio.

* *p* < .05.

### Links between quantitative variables, sociodemographic factors, and internalized MMM

Table 4 also presents results from linear regression models for two quantitative variables: the scale on personal discrimination and the item on vicarious discrimination. In these analyses, we followed the same structure as our logistic regression models. For personal discrimination, there were no significant results for demographic variables and internalized MMM. For vicarious discrimination, there were no significant differences by ethnic group and generational status. However, compared with men, women reported significantly more vicarious discrimination, and greater internalized MMM was significantly associated with less vicarious discrimination. Furthermore, we found that greater internalized MMM was linked to less vicarious discrimination over‐and‐above ethnic group, gender, and generational status.

## DISCUSSION

Utilizing a mixed methods approach, the current study provided a nuanced understanding of A/AA college students' experiences of discrimination, related to internalized MMM and sociodemographic factors, during the syndemic of COVID‐19 and our society's racial reckoning. The themes identified from our consensual qualitative approach demonstrated a range of experiences among A/AA during COVID‐19, from expressing fear and vigilance to denying and minimizing the pandemic's impact on anti‐Asian discrimination. Through a series of logistic and linear regression analyses, we also found that greater internalized MMM was linked to less reflections of vicarious discrimination, and that endorsement of qualitative themes and quantitative variables differed by Asian ethnic group, gender, and generational status.

Through our qualitative analysis of open‐ended responses about A/AA's experiences during COVID‐19, the most frequently endorsed theme was Vicarious Discrimination (30.4%). Many reflected on the negative impact of witnessing COVID‐related discrimination faced by fellow A/AA through social media, news platforms, and friends and family. Such experiences demonstrate how injustices committed against other members of the same racial group are collectively distressing and can lead to feeling fearful about being targeted next and preemptively engaging in efforts to avoid discrimination, which were experiences reflected in the Vigilance theme. Endorsement of the theme of Personal Discrimination was less frequent than Vicarious Discrimination but still evident in our findings (13.6%), as A/AA recounted their experiences of racial harassment and xenophobia during the pandemic. For our mixed methods purpose of triangulation, we also quantitatively measured A/AA's experiences of discrimination, and students who endorsed qualitative themes of Personal and Vicarious Discrimination and Vigilance were more likely to report higher levels of personal and vicarious discrimination.

The second most frequently endorsed theme was No Difference (26.3%), where students denied the impact of COVID‐19 on their racial discrimination experiences. Few studies have documented these types of reflections on the pandemic, where A/AA students explicitly stated that they did not experience racial discrimination or described their experiences as “Normal,” even “Fine,” and “Good.” Some students qualified that their lack of discriminatory experiences during COVID‐19 can be attributed to their ethnicity, particularly being South Asian, or their environments, which felt safer with a large amount of A/AA representation or other forms of diversity. Overall, our qualitative findings are consistent with extant literature documenting anti‐Asian racism and vigilance due to COVID‐19 (Chae et al., 2021; Hahm, Xavier Hall, et al., 2021; Zong et al., 2021), but also expands previous scholarship by highlighting a range of experiences among the A/AA community during the pandemic. This reflects the diversity within this broad racial group and rejects the perception that Asian Americans are a monolith, which is important to consider when addressing the unique needs of A/AA populations amidst the syndemic.

For the mixed method's purpose of expansion, we extended our investigation by putting qualitative themes around A/AA's experiences in conversation with a quantitative measure of internalized MMM. Results showed that students with higher levels of internalized MMM were less likely to endorse the Vicarious Discrimination theme. This finding suggests that internalizing the MMM is a barrier for A/AA to perceive and reflect on the pervasiveness of systemic anti‐Asian racism during COVID‐19. Consistent with racial triangulation theory (C. J. Kim, 1999), A/AA is uniquely positioned in the racial hierarchy as “problem‐free” minorities who do not experience structural oppression and internalizing individual‐level ideologies may have negative consequences on A/AA's awareness and willingness to challenge racial injustice, as well as implications for system‐level processes that maintain racism. Without understanding the structural forces that marginalize A/AA, it is unlikely for individuals to take action to eradicate anti‐Asian racism. Indeed, our findings are in line with previous studies demonstrating links between internalized MMM, less awareness of the structural nature of racism, and less own‐group activism and intergroup collective action (Ouch & Moradi, 2022; Tran & Curtin, 2017; Yi & Todd, 2021). It is also possible that A/AA college students with high internalized MMM are less likely to seek out or be exposed to anti‐Asian hate crimes through social media, or their friends and family similarly deny the pandemic's impact on widespread anti‐Asian racism; thus, community psychologists should specifically investigate how the various ecological contexts that A/AA are embedded in may shape their internalized MMM. Of note, we did not find associations between internalized MMM and the theme No Difference. However, it is important to consider how this theme primarily included a brief, one‐word response where it is uncertain whether they acknowledge the rise in anti‐Asian violence during COVID‐19 but just personally have not experienced racist incidents. Future research is needed to further examine the nuances in denying and minimizing structural barriers and discrimination experienced by A/AA.

Our study also examined associations between sociodemographic factors and A/AA's responses to COVID‐19. Specifically, investigating intersectional identities of ethnicity, gender, and generational status allowed us to understand multiple oppressive forces in the lives of A/AA and follow calls in the field to reject the homogenization of A/AA in research (Tseng & Lee, 2021). We found that South Asian students were more likely to endorse the No Difference theme and less likely to report Discrimination and Vigilance themes than East Asian students, but there were no significant differences between Southeast and East Asians. This suggests that the anti‐Chinese rhetoric fueled by the pandemic not only impacts East Asians but also Asian communities with phenotypical similarity, as well as reflects the racialized homogenization and collective targeting of Asian Americans (Cheng et al., 2021). However, these findings should be interpreted with caution, because our investigation of ethnic group differences could have been underpowered due to relatively small sample sizes. It is imperative that future scholarship examines the ethnic diversity in experiences of discrimination among A/AA with larger samples.

In general, results also showed that women and gender minorities were more likely to report experiences of vicarious discrimination and vigilance, which corroborates extant studies on the gendered nature of anti‐Asian racism during COVID‐19 (Jeung et al., 2021; Ren & Feagin, 2021). Our results on generational status also mirrored Liu et al.'s (2020) findings, where second‐generation immigrants were more likely to report discrimination and vigilance compared to international/first‐generation students. Of note, it is possible that these results were driven by the attitudes and experiences of international students, given that they comprised 72.4% of this group. It is important for future studies to distinguish between international students and first‐generation immigrant students, given that residency status can shape attitudes about and experiences of race and racism (Aggarwal & Çiftçi, 2021). More research is needed on factors such as length of time in the United States, acculturation levels, and awareness of structural racism that may differ by generational status and impact reporting of discriminatory experiences, as well as may shape the internalization process of the MMM. Finally, our study also found that greater internalized MMM, over‐and‐above sociodemographic factors, was associated with less vicarious discrimination. This finding is in line with scholarship demonstrating the pervasive nature of internalized oppression among A/AA with diverse, intersectional identities and its negative psychological and sociopolitical consequences (P. Y. Kim & Lee, 2014; Wong‐Pandoongpatt et al., 2022; Yi & Todd, 2021), and future research should examine the relationship between internalized MMM and anti‐Asian racism as it continues to transform in the U.S. context.

### Limitations

The current study had several limitations. First, we cannot infer directionality or establish causality, such as whether internalized MMM led to certain experiences during the syndemic. Future studies should utilize longitudinal methods to understand the development of internalized MMM and the lasting impact of COVID‐19 on their racial attitudes. Second, the generalizability of our results is limited to college students at a large Midwestern university, and different findings may have emerged for A/AA from different age groups and U.S. regions. Third, our quantitative measure of personal discrimination, although based on previously developed items, has not been widely tested, and their validity and reliability have not been established. Also, our quantitative measure of vicarious discrimination, which was developed for this study, was only one item. More scale development and psychometric work are needed to advance the assessment of personal and vicarious experiences of discrimination. Fourth, our use of one open‐ended question about A/AA's experiences during COVID‐19 with no follow‐up questions may have limited the potential depth and richness of the qualitative data. Finally, due to limited sample sizes, we did not test differences by specific ethnicities and used regional ethnic groupings, which may have glossed over important differences among A/AA. Thus, our investigation of regional ethnic group differences could have been underpowered, and future research should address these limitations by collecting larger sample sizes of A/AA ethnic groups.

### Implications for research and practice

The current study fills a critical gap in the field's understanding of the racialized experiences of A/AA and how internalized MMM shapes these experiences. Particularly given recent calls for community psychologists to uplift researchers with the cultural attunement and insight to study the complex experiences of BIPOC communities (Grills et al., 2022), the current study is an example of how alignment between researchers and communities of study can uncover important nuances in the experiences of these communities. Scholars should employ more in‐depth qualitative methods to further investigate the role of internalized MMM, as well as other forms of internalized oppression, in A/AA college students’ experiences amidst the syndemic. For example, future studies could interview A/AA students to understand how and why they adopt beliefs about their racial group as problem‐free, and how witnessing pandemic‐fueled anti‐Asian racism may shift their endorsement of the MMM. Furthermore, while we did not find a significant relationship between internalized MMM and the No Difference theme, future research is needed on how internalized oppression may shape A/AA college students' lack of awareness of the pandemic's impact on anti‐Asian racism. Future studies should extend our findings on sociodemographic factors by further utilizing intersectional approaches, such as examining the compounding effects of racism, sexism, xenophobia, ableism, and heterosexism during the syndemic. Finally, the rise in anti‐Asian racism has amplified grassroots social justice movements, including among college students; future research should examine how internalized MMM and experiences of discrimination connect with critical action within communities.

This study also has practical implications for psychologists, as we continue to navigate the syndemic of COVID‐19 and anti‐Asian racism. Our findings on the consequences of internalized MMM demonstrate the need for interventions that raise awareness of the pernicious effects of systemic racism among A/AA college students. For example, through online support groups and educational workshops on campus, A/AA students can process their experiences of anti‐Asian COVID‐19 racism and stress, learn about the historical and current impacts of the MMM in maintaining the U.S. racial hierarchy, and develop skills to engage in antiracism action and build solidarity among BIPOC (Cheng et al., 2021). Community psychologists in particular are well‐positioned to help design and implement such interventions that cultivate awareness of systemic racism and address internalized oppression as a means to build resilience among marginalized students (Watts et al., 1999). Our study also suggests that interventions for specific subgroups (e.g., second‐generation, East Asian women) are important to address the diversity within the A/AA campus community and may promote action through connection. Moreover, it is imperative that campus leaders and administrators publicly denounce anti‐Asian racism, implement systems to monitor discriminatory incidents, and provide spaces to listen to and support A/AA students (Tausen et al., 2020). Community psychologists, particularly those in positions of power and privilege, must engage in these types of systematic efforts to increase public consciousness about and take action against the pervasive and long‐standing damage of the MMM and anti‐Asian racism further exposed by COVID‐19.

This study highlights ways to better contextualize A/AA's marginalized experiences during the syndemic and furthermore are in line with community psychology's values in challenging systems of oppression. Our findings contribute to scholarship in community psychology by addressing a critical gap in examining A/AA's experiences of discrimination and have implications for future research and practice aiming to address internalized oppression and dismantle racial hierarchies.

## CONFLICT OF INTEREST

The authors declare no conflict of interest.
